# Effects of Melatonin on Antioxidant Capacity in Naked Oat Seedlings under Drought Stress

**DOI:** 10.3390/molecules23071580

**Published:** 2018-06-29

**Authors:** Wenying Gao, Yujing Zhang, Zheng Feng, Qingqing Bai, Jinjin He, Yingjuan Wang

**Affiliations:** State Key Laboratory of Biotechnology of Shannxi Province, Key Laboratory of Resource Biology and Biothchnology in Western China (Ministry of Education), College of Life Science, Northwest University, Xi’an 710069, China; 201620849@stumail.nwu.edu.cn (W.G.); 201520869@stumail.nwu.edu.cn (Y.Z); f7616287@163.com (Z.F.); 201731801@stumail.nwu.edu.cn (Q.B.); hjj18691403985@163.com (J.H.)

**Keywords:** drought stress, melatonin, naked oat seedling, antioxidant

## Abstract

Melatonin (*N*-acetyl-5-methoxytryptamine, MT) is a molecule with pleiotropic effects including antioxidant activity, regulated plant growth, development, and reduced environmental stress in plants. However, only a few studies have analyzed the effect of exogenous MT on drought stress in naked oat seedlings. Therefore, in this study, we studied the effects of exogenous MT on the antioxidant capacity of naked oat under drought stress to understand the possible antioxidant mechanism. The results showed that a pretreatment of 100 μM MT reduced the hydrogen peroxide (H_2_O_2_) and superoxide anion (O^2−^•) contents. MT also enhanced superoxide dismutase (SOD), peroxidase (POD), catalase (CAT), and ascorbate peroxidase (APX) activities in the leaves of naked oat seedlings under 20% PEG-6000 drought stress. MT upregulated the expression levels of the mitogen-activated protein kinases (MAPKs) *Asmap1* and *Aspk11*, and the transcription factor (TF) genes (except for *NAC*), *WRKY1*, *DREB2*, and *MYB* increased in drought with MT pretreatment seedlings when compared with seedlings exposed to drought stress alone. These data indicated that the MT-mediated induction of the antioxidant response may require the activation of reactive oxygen species (ROS) and MAPK, followed by triggering a downstream MAPK cascade such as Asmap1 and Aspk11, to regulate the expression of antioxidant-related genes. This study demonstrated that MT could induce the expression of MAPKs and TFs and regulate the expression of downstream stress-responsive genes, thereby increasing the plant’s tolerance. This may provide a new idea for MT modulation in the regulation of plant antioxidant defenses. These results provide a theoretical basis for MT to alleviate drought stress in naked oat.

## 1. Introduction

Naked oat (*Avena nuda* L.) is an annual herbaceous crop in the family *Poaceae*. Naked oat is a high nutritional value cereal [1] and is a unique food crop in Chinese alpine regions. Naked oat is extremely nutritious and medically valuable, and it is also used as both a food and a grass for animal husbandry production [1,2,3]. However, abiotic stressors directly limit the growth of naked oat, which restricts the effective use and development of the plant [4].

Abiotic stress greatly limits the growth and yield of plants. With the increase in global temperatures, the effect of drought stress is particularly prominent, and has become the main environmental factor limiting the growth and development of plants worldwide [5]. In general, drought stress causes a series of physiological and biochemical reactions such as stomatal closure, decreased chlorophyll content, decreased transpiration, and reduced antioxidant capacity [6]. Drought stress increases the production of ROS such as H_2_O_2_ and O^2−^•. Membrane lipid peroxidation is triggered when the balance between ROS accumulation and the free radical scavenging systems is disrupted, causing damage to the membrane system and leading to an imbalance in plant metabolism [7]. Mechanisms have developed in plants to combat adverse conditions (the main factor that limits plant productivity), which can prevent harmful damage [5], and the antioxidant system is one of the best ways plants resist stress. Plant antioxidant enzyme systems, such as SOD, POD, CAT, and APX scavenge ROS [8]. Similarly, ROS also activates or induces stress-related factors such as MAPK and TF, which further act as inducers or activators to activate response gene expression, thereby enhancing stress tolerance [9]. The increased expression and activity of these systems help maintain normal cellular metabolism and improve plant resistance to the stressor. Changes in external conditions activate the plant’s internal antioxidant system to maintain and balance normal physiological reactions, which directly affect the plant’s ability to resist adversity [10].

Melatonin (*N*-acetyl-5-methoxytryptamine, MT) is a small molecule neuroendocrine hormone. In animals, MT functions as a regulator of circadian rhythms [11], sleep, and the immune system [12], and has antitumor [13] and antioxidant activities [14,15,16,17]. MT was identified in plants for the first time by Dubbels et al. [18] and Hattori et al. [19]. Thereafter, MT has been found in a variety of plant species [20,21,22,23] with various biological functions such as the morphogenesis of organs [24], regulation of circadian rhythms, and, in flower development, the promotion of photosynthesis, fruit maturation, protection of chlorophyll [25,26], and delaying leaf senescence [27,28]. MT may also have antioxidant effects in plants. The antioxidant effects of MT have been confirmed in apple [29], rice [30], and grape [31]. Several studies have reported that MT enhances drought resistance in crops [32,33]. However, there is little research on antioxidant protection in naked oat. Therefore, in the present study, naked oat seedlings under drought stress were treated with MT to study the effects of MT on antioxidant enzyme activities and the expression levels of related resistance genes in leaves. The results will provide a basis for understanding the MT response mechanism during plant drought.

## 2. Results

### 2.1. Effect of Spraying 100 μM MT on Leaves of Naked Oat Seedlings under Drought Stress

**Figure 1:**
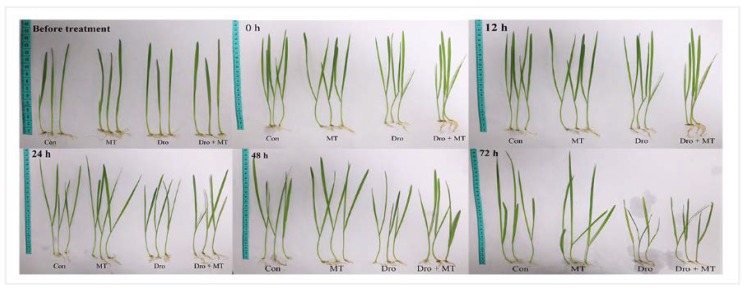
The performance of naked oat seedlings under normal and drought stress conditions. Con: seedlings grown in half-strength Hoagland solution; MT: seedlings pretreated with 100 μM MT, grown in half-strength Hoagland solution; Dro: seedlings grown in half-strength Hoagland solution plus 20% PEG-6000; Dro + MT: seedlings pretreated with 100 μM MT, grown in half-strength Hoagland solution plus 20% PEG-6000. Photographs were taken before melatonin pretreatment, and after drought stress with melatonin pretreatment at 0, 12, 24, 48, and 72 h.

**Figure 2:**
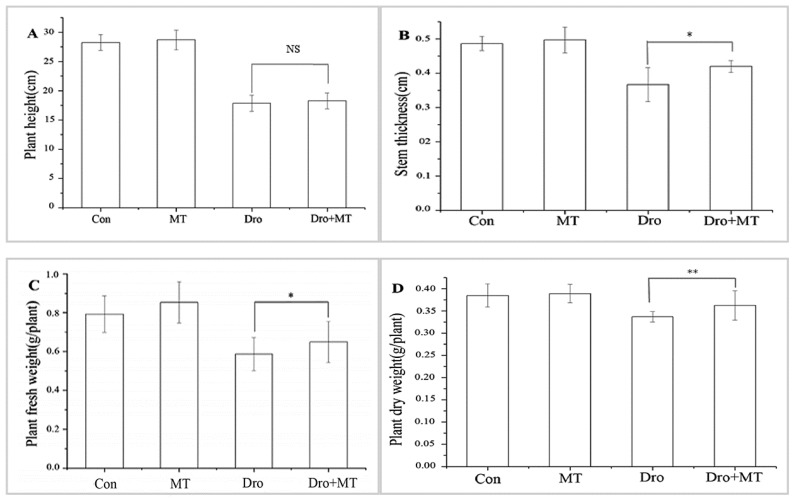
Effect of 100 μM melatonin on growth of naked oat seedlings. (**A**) Plant height; (**B**) Stem thickness; (**C**) Plant fresh weight; (**D**) plant dry weight. Values represent mean ± standard deviation (*n* = 3). Significant difference between drought with pretreatment MT and drought-treated. Asterisks; *, ** indicate *p* values, <0.05 and 0.01, respectively. NS; No significance. Control, untreated, Con; melatonin pretreatment, MT; drought, Dro; drought plus 100 μM melatonin pretreatment, Dro + MT.

The growth of naked oat seedlings was inhibited by drought stress, and 100 μM MT promoted seedling growth (Figure 1 and Figure 2). Under drought stress, plant height, stem thickness, plant fresh weight, and plant dry weight of naked oat seedlings were all inhibited, but that response was alleviated by adding MT. The increases for drought with MT-pretreated plants were 2.32% for plant height, 14.55% for stem thickness, 10.74% for plant fresh weight, and 7.57% for plant dry weight when compared with the drought group, respectively.

### 2.2. Effect of MT Pretreatment on Antioxidant Enzyme Activities of Naked Oat Seedlings under Drought Stress

**Figure 3:**
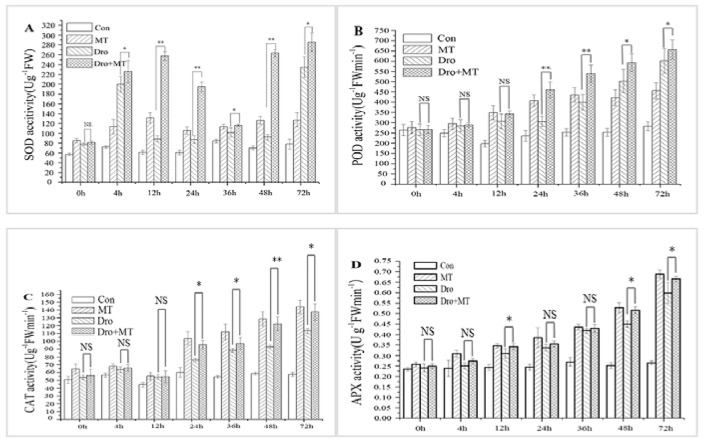
Effect of melatonin pretreatment on the antioxidant enzyme activities of naked oat seedlings under drought stress. (**A**) Super oxide dismutase (SOD); (**B**) peroxidase (POD); (**C**) catalase (CAT); (**D**) ascorbate peroxidase (APX). Values represent mean ± standard deviation (*n* = 3). Significant difference between drought with pretreatment MT and drought-treated. Asterisks; *, ** indicate *p* values, <0.05 and 0.01, respectively. NS; No significance. Drought plus melatonin pretreatment at times 0, 4, 12, 24, 36, 48, and 72 h.

Drought stress and the application of exogenous MT affected the antioxidant enzyme activities of the naked oat seedlings (Figure 3). The activities of SOD, POD, CAT, and APX in naked oat seedlings were significantly induced under drought stress. However, the 100 μM exogenous MT pretreatment increased the antioxidant enzyme activities of naked oat seedlings. When the stress duration was 1 day, 2 day, and 3 day, the average activities of SOD, POD, CAT, and APX significantly (*p* < 0.05) increased by 110.41%, 34.29%, 26.14%, and 10.35%, respectively, after imposing drought stress and the 100 μM MT pretreatment when compared with the drought group. The data showed that exogenous MT increased the activities of the major antioxidant enzymes in naked oat seedlings under drought stress.

### 2.3. Effect of MT Pretreatment on Changes in ROS in Naked Oat Seedlings under Drought Stress

**Figure 4:**
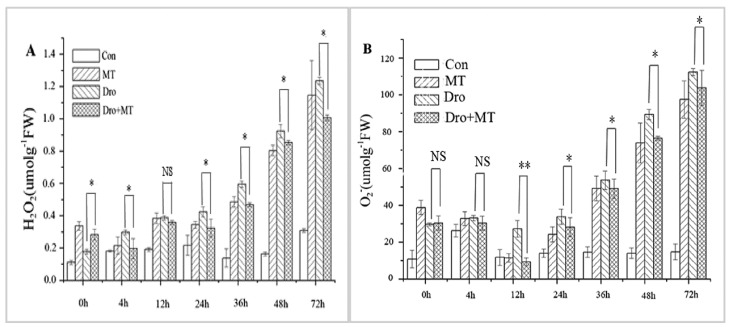
Effect of MT pretreatment on the changes of ROS in the naked oat seedlings under drought stress. (**A**) H_2_O_2_; and (**B**) O^2−^•. Values represent mean ± standard deviation (*n* = 3). Significant difference between drought with pretreatment MT and drought-treated. Asterisks; *, ** indicate *p* values, <0.05 and 0.01, respectively. NS; No significance. Drought plus melatonin pretreatment at times 0, 4, 12, 24, 36, 48, and 72 h.

H_2_O_2_ and O^2−^• are the two main forms of ROS produced under stress conditions [34]. H_2_O_2_ and O^2−^• contents increased in naked oat seedlings under drought stress and these increases were attenuated by pretreatment with MT (Figure 4). For instance, when compared with the control group, the H_2_O_2_ and O^2−^• contents in the naked oat seedlings significantly (*p* < 0.05) increased by 208.98% and 257.32%under drought stress, respectively. The accumulation of H_2_O_2_ and O^2−^• was alleviated by the MT pretreatment during drought stress, and decreased by 13.65% and 13.70%, respectively.

### 2.4. Effect of MT Pretreatment on MAPK Activity in Naked Oat Seedlings under Drought Stress

**Figure 5:**
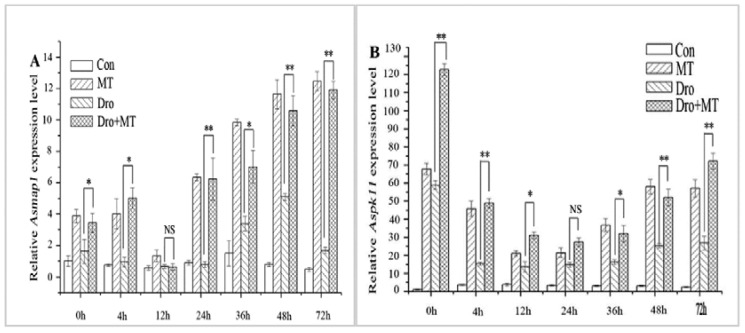
Effect of MT pretreatment on expression of MAPKs in leaves of naked oat seedlings. The relative expression of protein kinase genes *Asmap1* (**A**) and *Aspk11* (**B**). Values represent mean ± standard deviation (*n* = 3). Significant difference between drought with pretreatment MT and drought-treated. Asterisks; *, ** indicate *p* values, <0.05 and 0.01, respectively. NS; No significance. Drought plus melatonin pretreatment at times 0, 4, 12, 24, 36, 48, and 72 h.

The MAPK cascade is a signal transduction system that regulates plant growth and development in response to changes in stress [35]. *Asmap1* and *Aspk11* are MAPK genes of naked oat that were upregulated by drought stress. The expression levels of *Asmap1* and *Aspk11* genes were higher under drought stress when compared to those in the untreated control (Figure 5). However, applying the MT pretreatment to leaves noticeably upregulated this response (Figure 5). The two MAPK genes were upregulated at 4 h,12 h, 24 h, 36 h, 48 h, and 72 h, and the average relative expression levels of the genes were 6.30-fold and 29.45-fold higher than the drought stress alone group of seedlings.

### 2.5. Effect of MT Pretreatment on the Expression of Antioxidant-Related TF Genes in Naked Oat Seedlings during Drought Stress

**Figure 6:**
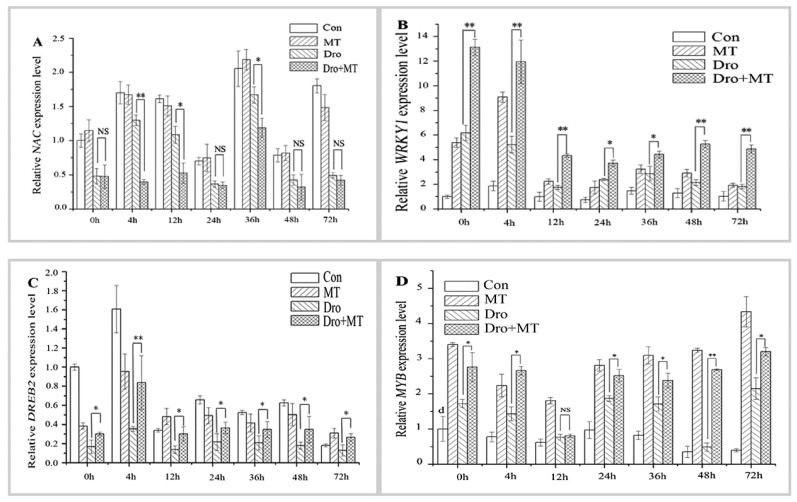
Effect of melatonin pretreatment on the expression of antioxidant-related TFs in leaves of naked oat seedlings. The relative expression of the related TFs genes *NAC* (**A**); *WRKY1* (**B**); *DREB2* (**C**); and *MYB* (**D**). Values represent mean ± standard deviation (*n* = 3). Significant difference between drought with pretreatment MT and drought-treated. Asterisks; *, ** indicate p values, <0.05 and 0.01, respectively. NS; No significance. Drought plus melatonin pretreatment at times 0, 4, 12, 24, 36, 48, and 72 h.

TFs regulate the expression of eukaryotic genes. When drought and high temperature stress conditions occur, the plant stimulates the expression of TFs to regulate the expression of downstream responsive genes that enhance the resistance to stress [36]. TFs such as NAC, WRKY, DREB, and MYB, which are closely related to the stress response in plants, help plants regulate their activities in adverse environments [37]. Drought stress affected the expression of antioxidant-related TFs in naked oat seedlings during the experiment (Figure 6). In our study, the average expression levels *MYB* and *WRKY1* in plants under drought stress were 0.74-fold and 2.00-fold higher than those in the control plants. However, after drought stress, the expression levels of *NAC* and *DREB2* decreased, and the expression levels were only 0.55-fold and 0.51-fold higher than those of the control group. Similarly, after drought with MT pretreatment, the expression levels *MYB* and *WRKY1* were higher than those of the drought-treated plants. However, the MT pretreatment clearly alleviated the suppression of the *DREB2* gene under drought stress in naked oat seedlings. The *NAC* gene appeared to be less affected under drought stress in the groups of naked oat seedlings with or without MT pretreatment.

## 3. Discussion

MT is an indoleamine that acts like an auxin (indole acetic acid) in plants to promote the growth of vegetative organs such as roots and leaves [38] as well as promote seed germination and seedling growth [39,40]. In the present experiment, we soaked naked oat seeds in different concentrations of MT. The effect of MT on seed germination rate, germination index, germination potential, and germination survival rate of the naked oat as a whole was manifested as promotion at a low concentration, and inhibition at a high concentration (Figure S1). The seed germination rate of the naked oat seeds was inversely related to the MT concentration. This result agreed with earlier studies in pea [41], but was contrary to the seed germination results of the same MT concentrations in *Arabidopsis thaliana* [42]. The differences in seed germination between the naked oat and *Arabidopsis thaliana* may be due to the differences in environmental conditions at the time of germination. The condition of *Arabidopsis thaliana* seeds germination was drought plus MT treatment, while the naked oat was only treated with MT. Moreover, the antioxidant potential of different plant seeds during germination differed [43], resulting in differences in germination between the naked oat and *Arabidopsis thaliana*.

Drought stress inhibited the growth of naked oat seedlings, whereas the MT pretreatment alleviated the inhibition of naked oat seedling growth caused by drought stress (Figure 3). Similarly, promotion of seedling growth has also been observed in soybean [44] and Bermuda grass (*Cynodon dactylon* (L.) Pers.) [45] under drought and salt stress conditions, but differed in the MT pretreatment concentration. Therefore, the effects of MT on plant growth under drought stress may be related to different sensitivities to MT by different plant species.

The production and elimination of ROS are unbalanced in plant cells under drought stress, and a large amount of ROS are produced, causing oxidative damage to plants [46]. MT induces antioxidant enzyme activities [47], which help to maintain a balance of ROS. The activities of the antioxidant enzymes SOD, POD, CAT, and APX were higher in the naked oat under drought stress with the MT pretreatment, and the enzyme activities increased as drought stress duration was prolonged (Figure 3). Similar studies in grape [46] and rapeseed [33] have shown that MT also enhanced antioxidant enzyme activities, thereby improving resistance to stress. Simultaneously, the drought treatment resulted in increased H_2_O_2_ and O^2−^• contents in naked oat leaves, and the MT pretreatment reduced the contents of H_2_O_2_ and O^2−^• (Figure 4), effectively inhibiting the increase in ROS in leaves of naked oat seedlings under drought stress. These results were similar to the effect of MT on wheat [47] and rapeseed [33]. These results showed that MT induced the increase in antioxidant enzyme activities in naked oat leaves to remove excess ROS and protect against oxidative stress in the plants.

The aerobic metabolites of cells are ROS. Stress increases the active oxygen yield and causes damage to cells. The H_2_O_2_ is an important signaling molecule for the plant stress response, and is widely involved in plant physiological and cross-resistance processes [8]. H_2_O_2_ acts as a second messenger in signal transduction of brassinosteroids (BRs) and abscisic acid (ABA)-induced plant resistance [48]. Xia et al. [48] speculated that BRs may induce the MAPK cascade reaction through the H_2_O_2_ pathway, thereby regulating the expression of antioxidant enzyme genes and promoting antioxidant enzyme activities, thereby enhancing the stress resistance of cucumber. In the present study, a large amount of H_2_O_2_ was produced under drought treatment in the leaves of the naked oat. H_2_O_2_ acted as a second messenger to activate the downstream MAPK cascade and upregulated the expression of antioxidant-related TFs genes, thereby enhancing naked oat seedling tolerance (Figure 4, Figure 5 and Figure 6). MT has a similar mechanism to BRs. MT pretreatment enhanced antioxidant enzyme activity and upregulated the expression of MAPKs and TFs genes (Figure 3, Figure 4, Figure 5 and Figure 6). This further confirmed the two effects of MT: MT scavenged ROS by enhancing antioxidant enzyme activity; (ii) MT might also induce MAPK cascade through the H_2_O_2_ pathway, thus improving the drought tolerance of naked oat seedlings. These results may provide new evidence to clarify the underlying mechanism of MT.

The MAPK cascade pathway responds to various biotic and abiotic stressors in plants, such as bacteria, high salinity, drought, and oxidative stress [49,50,51]. Phosphorylate MAPK acted as a bridge between upstream receptors and downstream transcription factor genes, and enhanced expression of the transcription factors *NAC*, *WRKY1*, *DREB2*, and *MYB* of naked oat. Activated TFs further regulated the expression of downstream responsive genes, thereby enhancing drought tolerance. In *Arabidopsis*, flagellin flg22 triggered the complete MAPK cascade and MEKK1-MKK4/5-MAPK3/6 activated the expression of the downstream gene *WRKY22/29*, which enhanced plant resistance to bacterial and fungal pathogens [52]. In rice, OsMAPK3 phosphorylates SP sites of OsWRKY30, enhancing tolerance to drought stress [53]. In the present study, Asmap1 and Aspk11 are two MAPKs in naked oat that were induced under drought with and without MT. The relative expression levels of *Asmap1* and *Aspk11*, which were involved in regulating the MAPK cascade through the H_2_O_2_ pathway, were significantly (*p* < 0.05) upregulated in response to drought stress. However, their relative expression was higher in drought plus MT plants. Thus, MT likely induced the MAPK cascade through the H_2_O_2_ pathway, which further enhanced the drought tolerance of naked oat seedlings.

**Figure 7:**
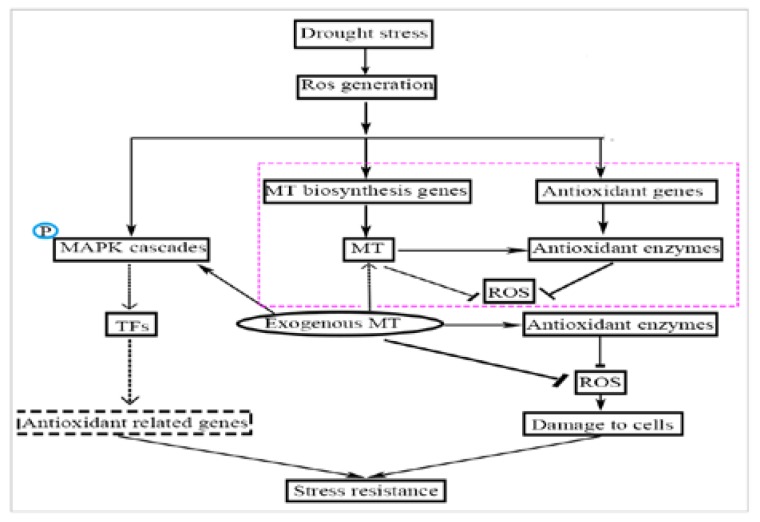
A schematic plot showing the potential mechanism of melatonin-mediated alleviation of drought-induced oxidative stress in naked oat seedlings [36,64].

TFs regulate the expression of eukaryotic genes, especially when plants are exposed to stress, such as drought or high temperature. TFs regulate the expression of downstream resistance genes, enhancing the plant’s resistance to stress [36]. The TFs that participate in plant stress resistance include NAC, WRKY, MYB, and DREB [37]. The rice NAC family (such as OsNAP, OsNAC22, SNAC1, and SNAC3) is induced by drought or ABA, which enhances drought resistance [54,55,56] and heat resistance [57] through the ABA pathway. The WRKY transcription factor family (LtWRKY21) in shrub plants enhances drought resistance though the ABA signal transduction pathways by enhancing the expression of downstream genes [58]. Overexpression of the WRKY transcription factor genes (*OsWRKY45* and *WRKY57*) in *Arabidopsis* upregulates ABA levels and increases drought tolerance [59,60]. Thus, the mechanism of MT-induced drought tolerance in naked oat may also involve the ABA signaling pathway, which needs further study. Studies have confirmed that the overexpression of the *OsWRKY45* and *WRKY57* genes is affected by ROS [61]. However, drought treatment decreased expression of the transcription factors *GmNAC2* and *GhWRKY*17 in tobacco, but induces the production of ROS [62,63]. In the current study, after drought with and without MT pretreatment, the expression levels of *WRKY1* and *MYB* were significantly (*p* < 0.05) upregulated (Figure 6B,D), but there was no significant change in the expression level of *NAC* (Figure 6A). Compared with drought alone, the expression level of *DREB2* was significantly (*p* < 0.05) upregulated (Figure 6C) in drought with MT-pretreatment. These results suggest that the TFs family NAC, WRKY1, DREB2, and MYB may be involved the MAPK cascade through the H_2_O_2_-induced signaling pathways in the stress response of naked oat. In summary, it is speculated that MT enhanced the drought stress response in naked oat seedlings (Figure 7). MT promoted the production and accumulation of ROS in cells. As the concentration of ROS in cells increased, MT acted as antioxidants to scavenge ROS and enhance antioxidant enzyme activities. The generated ROS induced or activated the downstream MAPKs and TFs, and regulated the expression of downstream resistance genes, which enhanced drought tolerance. Thus, appropriate concentrations of MT could positively stimulate stress resistance in naked oat, but further study is required to fully understand the response mechanism and signaling pathways of MT under drought stress in naked oat.

## 4. Materials and Methods

### 4.1. Plant Materials

Naked oat seeds of the ‘Jin Yan No. 2’ cultivar (*A. nuda* L.) were provided by the Provincial Key Laboratory of Biotechnology of Shaanxi Province, Northwest University, Xi’an 710069, Shaanxi Province, China. They were stored at 4 °C under dry conditions, before the experiments started.

### 4.2. Seed Germination Conditions

Naked oat seeds were surface-sterilized with a 75% (*v*/*v*) alcohol solution for 15 s, washed two to three times in distilled water, sterilized with 1% HgCl_2_ (*m*/*v*) for 7 min, and washed again five or six times in distilled water. The seeds were soaked in a 100 μM concentration for 12 h, then placed on 14-cm diameter Petri dishes (40 seeds per dish) with three layers of filter paper in a dark growth chamber (23–25 °C/16–18 °C day/night) for germination. All seeds were fertilized daily with half-strength Hoagland solution (pH 6.5 ± 0.1). On the basis of the state of naked oat germination and growth in our preliminary studies, we selected a concentration of 100 μM MT (Figure S1).

### 4.3. MT Pretreatment

After the emergence of the radicle, seedlings were shifted to hydroponic conditions containing half-strength Hoagland solution. After five days, half of the seedlings were shifted to half-strength Hoagland solution, while the other half was shifted to half-strength Hoagland solution containing 100 μM MT. A total of 0.05807 g of MT was dissolved in ethanol absolute at a concentration of Twenty-five mmol/L and stored at −20 °C. Twenty-five mmol/L of MT was further diluted to 100 μM. The naked oat leaves were sprayed with 100 μM of MT for the first time at 8 p.m. and then sprayed once every day. Exogenous 100 μM MT was sprayed three times and the experimental period was six days. During the MT pretreatment, seedlings were selected and placed in a growth room with a relative humidity of 75%, a 14L:10D photoperiod, and day/night temperatures of (25 ± 1) °C/(17 ± 1) °C.

### 4.4. Drought-Stress Treatment

After two days of MT pretreatment, a 20% (*m*/*v*) poly-ethylene glycol-6000 (PEG-6000) drought stress treatment was applied. Naked oat leaves were harvested at 0, 4, 12, 24, 36, 48, and 72 h after the drought stress treatment, rapidly frozen in liquid nitrogen, and stored at −80 °C for further analysis. The experiment was divided into four groups of experiments with 60 seedlings each. According to the growth states of naked oat seedlings under different drought stress conditions, and the effect of spraying different concentrations of MT on the leaves of naked oat seedlings under 20% PEG-6000 drought stress (Figures S2–S4), the concentration of MT (100 μM) and PEG-6000 (20%) was selected based on our results.

### 4.5. Determination of Various Indicators

#### 4.5.1. Calculation of Plant Height, Stem Thickness, Plant Fresh Weight, and Plant Dry Weight

After treatment in 20% PEG-6000 for three days, seedlings were randomly selected from each dish and plant height, stem thickness, plant fresh weight, and plant dry weight were measured. The height of the plant was the length of the seedling, which was the linear distance from the base of the radicle to the top of the blade. The fresh/dry weight of the seedling was the total fresh/dry weight of the entire seedling. Naked oat seedlings were harvested, then washed with tap water and rinsed three times with distilled water, gently wiped dry with a paper towel and their fresh weight (FW) was determined rapidly. Then, the seedlings were dried at 105 °C for 30 min, and dried at 80 °C for 24 h to measure their dry weight (DW). Three parallel experiments were performed simultaneously.

#### 4.5.2. Enzyme Extraction and Assay

All enzymes were extracted by grinding 0.2 g of fresh leaves with 3 mL of ice-cold 50 mM phosphate buffer (pH 7.8) containing liquid nitrogen using a chilled mortar and pestle. The homogenate was centrifuged at 11,000 rpm/min for 20 min at 4 °C, and the supernatant was used for the specific enzyme activity assays. The activity of SOD (EC 1.15.1.1) was determined by the method described by Giannopolitis and Ries. [65] through measuring its ability to inhibit the photochemical reduction of nitro blue tetrazolium (NBT) in a spectrophotometer at 560 nm, the POD (EC 1.11.1.7) activity analysis was calculated using guaiacol in a spectrophotometer at 470 nm according to the method described by Cakmak and Marschner [66], CAT (EC 1.11.1.6) activity was assessed in spectrophotometer at 240 nm according to Hamurcu et al. [67], and APX (EC 1.11.1.11) activity was measured in a spectrophotometer at 290 nm by the method described by Nakano and Asada [68].

#### 4.5.3. Determination of H_2_O_2_ and O^2−^•

Production of H_2_O_2_ was estimated following Yu et al. [69]. Naked oat leaves were extracted by grinding 0.2 g of fresh leaves with 3 mL of ice-cold 50 mM phosphate buffer (pH 6.5) containing liquid nitrogen using a chilled mortar and pestle. The homogenate was centrifuged at 11,500 rpm/min for 10 min at 25 °C, and the supernatant was used for the H_2_O_2_ content assay in a spectrophotometer at 410 nm.

O^2−^•—was measured through a method described by Velikova et al. [70]. Leaves were extracted by grinding 0.3 g of fresh leaves with 3 mL of ice-cold 50 mM potassium phosphate buffer (pH 7.8) containing liquid nitrogen using a chilled mortar and pestle. The homogenate was centrifuged at 5000 rpm/min for 20 min at 25 °C, and the supernatant was used for the O^2−^• content assay in a spectrophotometer at 530 nm.

#### 4.5.4. Quantitate Real Time-Polymerase Chain Reaction (qRT-PCR) Analysis

The drought-tolerant related gene sequences of oat *Asmap1* (X79993.1), *Aspk11* (X79992.1), NAC (KU886332.1), *WRKY1* (AF140554.1), *DREB2* (EF672101.1), and *MYB* (AJ133638.1) were searched on NCBI; they were compared with the gene sequences of other species by BLAST, and the homologous sequences of the genes from several species were downloaded. Degenerate primers were designed and the conserved regions sequences were amplified (≤800 bp) and verified using MEGA software (Center for Evolutionary Medicine and Informatics, The Biodesign Institute, McAllister Ave, Tempe, AZ, USA). The qRT-PCR primers were designed using Primer 5.0 software (Premier Biosoft, Palo Alto, CA, USA) (Table 1).

The TRIzol reagent (Invitrogen, Carlsbad, CA, USA) method was used to extract total RNA from naked oat leaves. First-strand cDNA was synthesized using the PrimeScriptTM RT reagent kit with the gDNA Eraser (Takara, Shiga, Japan) according to the manufacturer’s instructions. qRT-PCR was performed on a Bio-Rad CFX96 Real-Time PCR System (Bio-Rad, Hercules, CA, USA) using FastStart Essential DNA Green Master (Tiangen, Beijing, China). The procedure was as follows: 95 °C for 10 min, one cycle, 40 cycles of 95 °C for 10 s, and 60 °C for 30 s. Finally, the melting curves were performed to confirm the specificity of the primers again by heating up the products from 60 °C to 95 °C. The *Actin* (KP257585.1) housekeeping gene was used to normalize the relative expression levels of the candidate genes. Three independent biological replications were performed for each experiment. The relative gene expression levels were calculated according to the 2^−ΔΔ*C*t^ method and presented as fold changes.

### 4.6. Statistical Analysis

The experiments were divided into the control untreated group (Con), melatonin-treated (MT), drought-treated group (Dro), and drought-treated group with melatonin (Dro + MT). All experiments were repeated three times, and mean values were presented with standard deviations. One-way analysis of variance (ANOVA) was according to Duncan’s test by SPSS 20.0 (IBM, Armonk, NY, USA). The difference was considered to be statistically significant when *p* < 0.05. Student’s *t*-test was also used to analyze the significant differences between drought with pretreatment MT. Data were plotted using Origin8.0 (OriginLab, Hampton, MA, USA) and graphs were edited in Photoshop CS5 (Adobe, USA).

## Figures and Tables

**Table 1:** Drought-tolerance relative genes and primers used in real-time qPCR analysis.

Gene Name	Sense Primer	Anti-Sense Primer
*NAC*	GGAGTCGGAGATCGTGGACACC	TGGATGTCGTCGTAGCTGAGGTC
*DREB2*	ATACCGTGGTGTGAGGCAG	CGAGATACGAGAAGGAGGA
*WRKY1*	GGCGTCCTCCTTCCTCCAGTC	CCTCGTATGGCGTGCTGAAGC
*MYB*	GAACCAGCAGCCGTCTGTGAG	GCAGGAGCGGTGGATTCAGTG
*Asmap1*	CATCCGCTCCAACCAAGAACTCTC	TACTCCGTCATCATGTCGCTCTCC
*Aspk11*	GGTCCATACCCCCACAGA	TAGTCCAACAGCCTCATT
*Actin*	ATGTTGCCATCCAGGCTGTG	TAAGTCACGTCCAGCGAGGT

## Supplementary Materials

The following are available online, Figure S1: Effect of different MT concentrations on germination of naked oat seeds, Figure S2: Effect of spraying different concentrations of MT on the growth of naked oat seedlings, Figure S3: Effect of different concentrations of drought treatment on the growth of naked oat seedlings, Figure S4: Effect of spraying different concentrations of MT treatment on the growth of naked oat seedlings under 20% PEG-6000 drought stress.

## Abbreviations

MT	Melatonin
H_2_O_2_	Hydrogen peroxide
O^2−^•	Superoxide anion
SOD	Superoxide dismutase
POD	Peroxidase
CAT	Catalase
APX	Ascorbate peroxidase
PEG-6000	Poly-ethylene glycol-6000
MAPKs	Mitogen-activated protein kinases
TF	Transcription factor
ROS	Reactive oxygen species
ABA	Abscisic acid
BRs	Brassinosteroids
