# Supplementary material for: Effects of Melatonin on Antioxidant Capacity in Naked Oat Seedlings under Drought Stress
Source: Molecules. 2018 Jun 29;23(7):1580. doi: 10.3390/molecules23071580 (PMC6099629; doi:10.3390/molecules23071580)
Supplement: Supplementary file 1 [file molecules-23-01580-s001.pdf]

Supplementary Materials

Effects of Melatonin on Antioxidant Capacity in Naked Oat Seedlings under Drought Stress

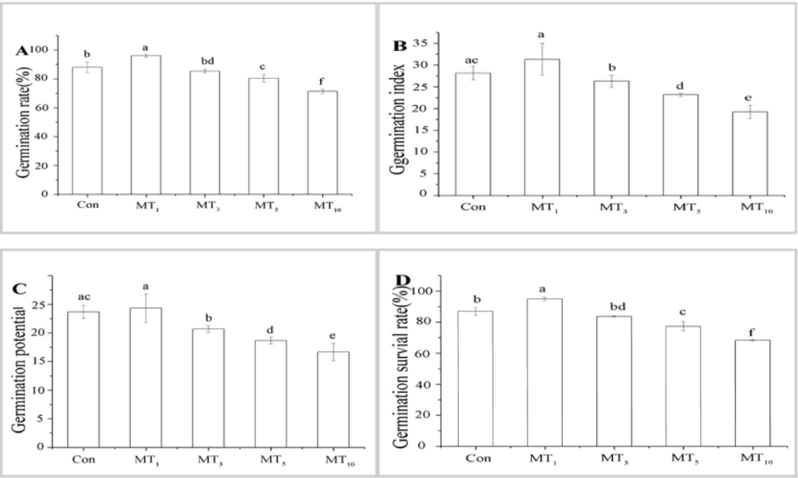

Figure S1. Effect of different MT concentrations on germination of naked oat seeds. (**A**) Germination rate; (**B**) germination index; (**C**) germination potential; (**D**) germination survival rate. Values represent mean ± standard deviation (n = 3). Different letters (a, b, c, d, e) on the bars indicate significant differences ($p < 0.05$) between different treatments according to Duncan's multiple range test comparison. Control seeds without melatonin pretreatment, Con; seeds pretreated with MT at concentrations of 100 μM (MT_1), 300 μM (MT_3), 500 μM (MT_5), and 1,000 μM (MT_{10}).

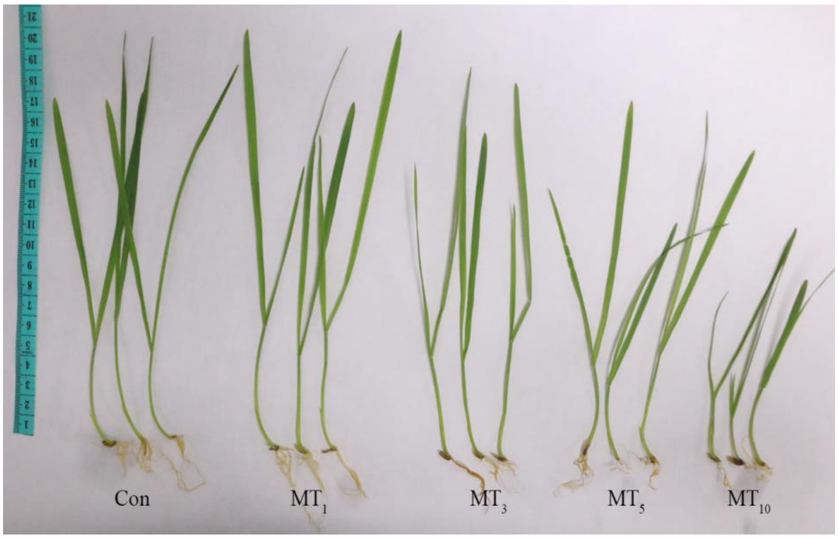

Figure S2. Effect of spraying different concentrations of MT on the growth of naked oat seedlings. Photograph was taken after spraying the MT for 1 day. Control seedlings without MT pretreatment, Con; seedlings sprayed with MT at concentrations of 100 μM (MT_1), 300 μM (MT_3), 500 μM (MT_5), and 1,000 μM (MT_{10}).

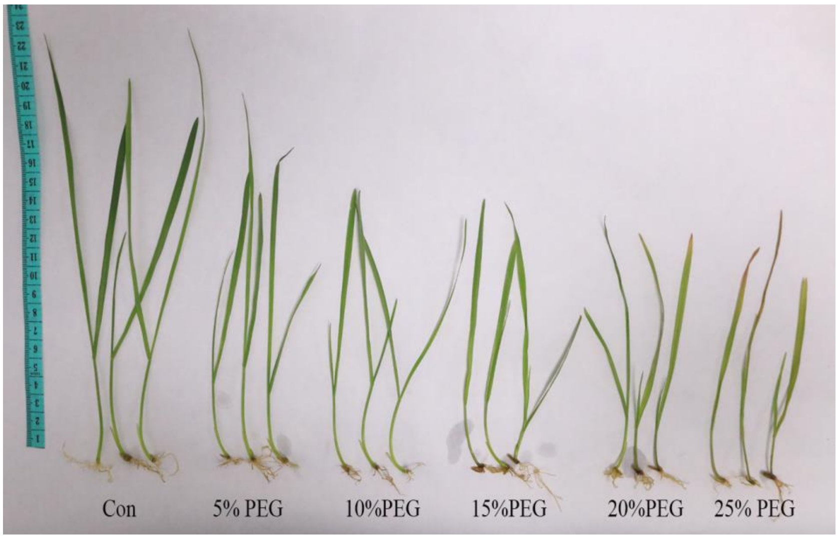

Figure S3. Effect of different concentrations of drought stress treatment on the growth of naked oat seedlings. Photograph was taken after drought for 1 day. Control seedlings without drought pretreatment, Con; seedlings pretreated with PEG-6000 at concentrations of 5 % (5 % PEG), 10 % (10 % PEG), 15 % (15 % PEG), 20% (20 % PEG), and 25% (25 % PEG).

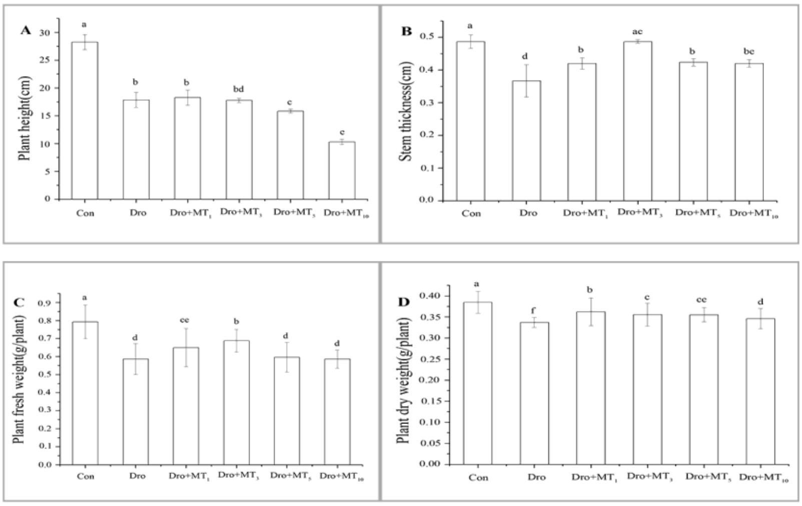

Figure S4. Effect of spraying different concentrations of MT on the growth of naked oat seedlings under 20% PEG-6000 drought stress. (**A**) Plant height; (**B**) stem thickness; (**C**) plant fresh weight; (**D**) plant dry weight. Values represent mean ± standard deviation (n = 3). Different letters on the bars indicate significant differences ($p < 0.05$) between different treatments according to Duncan's multiple range test comparison. Control-untreated, Con; drought (Dro) plus the MT pretreatment at concentrations of 100 µM (Dro + MT_1), 300 µM (Dro + MT_3), 500 µM (Dro + MT_5), and 1,000 µM (Dro + MT_{10}).
